# Pediatric Uveitis in a Tertiary Referral Center in East China: Clinical Patterns and Visual Outcomes

**DOI:** 10.1155/joph/5015614

**Published:** 2024-11-21

**Authors:** Boya Lei, Xianjin Zhou, Ruiping Gu, Qinmeng Shu, Xinyi Ding, Rui Jiang, Qing Chang, Gezhi Xu, Haimei Liu, Li Sun, Min Zhou

**Affiliations:** ^1^Eye Institute and Department of Ophthalmology, Eye and ENT Hospital, Fudan University, Shanghai, China; ^2^Shanghai Key Laboratory of Visual Impairment and Restoration, Fudan University, Shanghai, China; ^3^NHC Key Laboratory of Myopia and Related Eye Diseases, Fudan University, Shanghai, China; ^4^Key Laboratory of Myopia and Related Eye Diseases, Chinese Academy of Medical Sciences, Shanghai, China; ^5^Department of Ophthalmology, Shanghai Children's Medical Center, Shanghai Jiao Tong University School of Medicine, Shanghai, China; ^6^Harvard Retinal Imaging Lab, Department of Ophthalmology, Massachusetts Eye and Ear, Harvard Medical School, Boston, Massachusetts, USA; ^7^Department of Rheumatology, Children's Hospital of Fudan University, Shanghai, China

## Abstract

**Background:** To describe the clinical patterns and visual outcomes of pediatric uveitis at a tertiary referral center in East China.

**Methods:** Retrospective case series. Clinical records of patients with pediatric uveitis who presented between January 2014 and July 2021 were reviewed.

**Results:** The children included (*n* = 283; 146 females, 137 males) had a mean age at presentation of 10.6 ± 3.5 years. There was a predominance of chronic (62.9%), noninfectious (81.3%) disease, and anterior uveitis was the most common uveitis type (54.8%). Idiopathic chronic anterior uveitis (17.3%) and juvenile idiopathic arthritis (JIA)–associated anterior uveitis (16.3%) were the most common noninfectious types; ocular toxocariasis (14.8%) and viral retinitis (1.4%) were the most common infectious etiologies. Ocular complications were observed in 53.0% of patients during follow-up. Systemic immunosuppressive therapy was administered to 66.8% of patients, 67.2% of whom required immunosuppressive drugs and/or biological agents (127/189 children). Surgical treatment was conducted in 38 (13.4%) patients. Improvement or preservation of visual acuity was observed in 95.2% of patients for whom follow-up visual acuity was recorded (179/188 patients).

**Conclusions:** Pediatric uveitis was predominantly chronic and noninfectious, with anterior involvement. Systemic therapy was required by most patients, and most eyes showed improved visual acuity.

## 1. Introduction

Uveitis is less common in children than in the adult population and accounts for only 5%–10% of all cases [1]. Its prevalence is estimated to be about 30 cases in 100,000 children [2]. The etiologies of uveitis in children are infectious or noninfectious. The symptoms differ from those in adults, and more patients are asymptomatic [3]. Examining young children of preverbal age is difficult, which makes the diagnosis and treatment of pediatric uveitis challenging. Given the characteristics of the affected population and the disease features, children with uveitis are more likely to suffer from chronic and severe inflammation [4, 5]. Because children are in a critical period of visual development, the accurate diagnosis and timely treatment of uveitis are very important for their future visual function and quality of life [6]. Therefore, familiarity with the etiological classification of uveitis in children is essential if ophthalmologists are to diagnose uveitis accurately.

Many reports worldwide have focused on the clinical patterns of pediatric uveitis, which vary according to its ethnic and geographic distributions [7–9]. In the United States, more than 90% of pediatric uveitis is noninfectious, and juvenile idiopathic arthritis (JIA)–associated anterior uveitis is the most common etiology (around 30%) [10], whereas in Colombia, the most common etiology is infection (58.7%) caused by toxoplasmosis (45.1%) [11].

Although studies focusing on the clinical patterns of pediatric uveitis have been published in several Asian countries, including India, Japan, and Singapore [8, 12, 13], there are limited data on these patterns in China. In the present study, we aim to describe the clinical patterns, treatment modalities, and visual outcomes of pediatric uveitis in a tertiary referral center in East China.

## 2. Materials and Methods

In this observational retrospective case series, we examined the clinical records of all subjects with a diagnosis of pediatric uveitis who visited our uveitis clinic between January 2014 and July 2021. The selection criterion for pediatric uveitis was the onset of uveitis before the age of 16 years.

Demographic and clinical data were collected, including sex, age at onset of uveitis, laterality and chronicity of uveitis, etiology, and any associated systemic disease. Ophthalmological characteristics were also collected, including the best corrected visual acuity (BCVA) at presentation and the last follow-up, anatomical distribution of uveitis, type of treatment, and ocular complications. Visual acuity was recorded with Snellen visual acuity ratios, whereas the logarithm of the minimum angle of resolution (logMAR) was used for statistical analyses. In patients with bilateral involvement, the analysis of ocular complications, ocular surgery, and visual outcomes was based on the eye with the worse visual acuity.

Blood tests (complete blood cell count, erythrocyte sedimentation rate, autoantibodies, human leukocyte antigen [HLA] typing, serum angiotensin-converting enzyme, antibodies against infective agents, tuberculosis gamma interferon release assay, genetic testing) and intraocular fluid tests (antibodies against infective agents, polymerase chain reaction [PCR] for pathogen nucleic acids) were performed according to the suspected type of uveitis, as well as auxiliary ophthalmic examinations (B-scan ultrasonography, ultrasound biomicroscopy, optical coherence tomography, fluorescein, and indocyanine green angiography), and systemic imaging studies (computed tomography scans of chest or orbits, joint radiography).

Uveitis was classified according to the Standardization of Uveitis Nomenclature [14]. Uveitis attributed to specific ocular causes was diagnosed using the current diagnostic criteria [15–19]. Uveitis attributed to systemic disease was confirmed by the clinical features of the patients and diagnostic tests, in collaboration with pediatric rheumatologists or infectious disease physicians, and according to established sets of criteria [20–27]. “Idiopathic uveitis” referred to cases that could not be attributed to any specific ocular cause or underlying systemic disease. Retinal vasculitis was also included in this study and classified as “posterior uveitis” for analysis and discussion.

Continuous data were described as means ± standard deviations. Categorical data were described as absolute and relative frequencies in each category. Nonparametric statistical tests (Wilcoxon's test, Mann–Whitney *U* test) were used for data that were positively skewed on histograms. *p* values of < 0.05 were considered to indicate statistical significance. SPSS for Windows, version 17.0 (SPSS Inc., Chicago, IL, USA), was used for all statistical analyses.

The study was conducted in accordance with the tenets of the Declaration of Helsinki and was approved by the Institutional Review Board of the Eye and ENT Hospital of Fudan University, Shanghai, China (Ethics No. 2020142). Informed consent was obtained from all respective parents or guardians of the participants.

## 3. Results and Discussion

### 3.1. Baseline Characteristics

A total of 283 consecutive pediatric patients were included in the present study. The male-to-female ratio was 1:1.07 (137 boys [48.4%] and 146 girls [51.6%]). The mean age at presentation was 10.6 ± 3.5 years (range: 3–16). Ninety-three patients (35 boys and 58 girls) were ≤ 8 years old, and 190 patients (102 boys and 88 girls) were > 8 years old. At the time of the survey, 140 (49.5%) patients had bilateral involvement and 143 (50.5%) had unilateral involvement. Chronic uveitis was observed in most patients (178 patients, 62.9%), followed by acute uveitis (83 patients, 29.3%) and recurrent uveitis (22 patients, 7.8%).

Anterior uveitis was the most common type and was observed in 155 (54.8%) patients, followed by panuveitis in 72 (25.4%) patients, posterior uveitis in 43 (15.2%) patients, and intermediate uveitis in 13 (4.6%) patients. Intraocular inflammation was associated with an infectious agent in 53 (18.7%) patients, whereas a noninfectious etiology was diagnosed in 230 (81.3%) patients. A specific ocular diagnosis was made in 61 (21.5%) patients, a systemic disease association was found in 80 (28.3%) patients, and the remaining 142 (50.2%) patients were classified with idiopathic uveitis because no specific cause was found. The demographic and clinical characteristics of the patients included are shown in Table 1.

### 3.2. Etiology

When uveitis was associated with an infectious agent, ocular toxocariasis was the leading cause (42 patients, 79.2%), followed by viral retinitis (four patients, 7.5%), ocular toxoplasmosis (three patients, 5.6%), and tuberculosis (two patients, 3.8%). In patients with noninfectious uveitis, chronic anterior uveitis was the leading cause (95 patients, 41.3%—including 49 patients with an idiopathic etiology and 46 patients with JIA-associated anterior uveitis), followed by idiopathic acute anterior uveitis (47 patients, 20.4%) and idiopathic retinal vasculitis (19 patients, 8.3%) (Table 2).

When uveitis had a specific ocular diagnosis, ocular toxocariasis was the leading cause (42 patients, 68.9%), followed by Vogt–Koyanagi–Harada syndrome (six patients, 9.8%) and acute retinal necrosis (four patients, 6.6%). When uveitis was associated with a systemic disease, JIA-associated anterior uveitis was the leading cause (46 patients, 57.5%), followed by Behçet's syndrome (12 patients, 15.0%) and HLA-B27-associated anterior uveitis (six patients, 7.5%). Chronic anterior uveitis was the leading cause of idiopathic uveitis (49 patients, 34.5%), followed by acute anterior uveitis (47 patients, 33.1%) and retinal vasculitis (19 patients, 13.4%) (Table 3).

### 3.3. Complications

**Figure 1:**
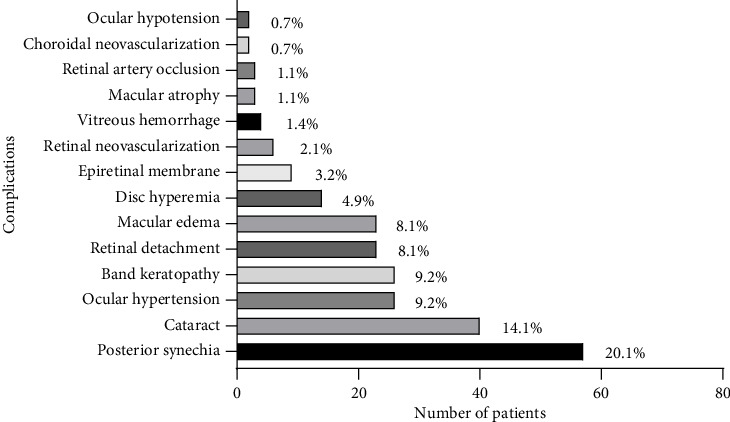
Complications observed throughout follow-up (numbers of patients).

One hundred and fifty (53.0%) patients suffered from at least one ocular complication in either eye during the follow-up period. The most common ocular complications included posterior synechia (57 patients, 20.1%), cataract (40 patients, 14.1%), ocular hypertension (26 patients, 9.2%), and band keratopathy (26 patients, 9.2%). The ocular complications observed throughout the follow-up period are shown in Figure 1.

### 3.4. Treatment Modalities and Visual Outcomes

The ophthalmic interventions of the patients were also reviewed. Eighty-two (29.0%) patients were treated with topical steroids, including eye drops or periocular injections. One hundred and eighty-nine (66.8%) patients received systemic immunosuppressive therapy, including only systemic steroids (62 [21.9%] patients), conventional immunosuppressive drugs (83 [29.3%] patients), and biological agents for uveitis refractory to conventional immunosuppressive agents (44 [15.5%] patients). Anti-infective agents were administered to 12 (4.2%) patients with an infective etiology.

Surgical treatments were administered in 38 (13.4%) patients, with 18 (6.4%) patients undergoing cataract surgery, 9 (3.2%) patients vitrectomy, and five (1.8%) patients intravitreal injections.

In eyes that received no surgical treatment, visual acuity improved from 0.478 ± 0.491 (Snellen equivalent 20/60) to 0.194 ± 0.410 (Snellen equivalent 20/31) after a follow-up period of 12.1 ± 11.0 months (*p* < 0.001); in those eyes that underwent surgical treatment, visual acuity improved from 1.591 ± 0.846 (Snellen equivalent 20/780) to 0.899 ± 0.861 (Snellen equivalent 20/159) after a follow-up period of 19.4 ± 13.9 months (*p*=0.001). The details of treatment modalities and visual outcomes are shown in Tables 4 and 5, respectively.

Among all patients with uveitis of any etiology and location, the visual acuity of 241 patients was recorded at presentation and that of 188 patients was recorded at follow-up. At presentation, 29.1% of patients (*n* = 70) had visual acuity ≤ 20/200; 26.1% of patients (*n* = 63) had visual acuity between 20/50 and 20/200; and 44.8% of patients (*n* = 108) had visual acuity > 20/50. At the last follow-up, 10.1% of patients (*n* = 19) had visual acuity ≤ 20/200; 16.0% (*n* = 30) had visual acuity between 20/50 and 20/200; and 73.9% (*n* = 139) had visual acuity > 20/50. Therefore, visual acuity improved in 166 (88.3%) patients, was unchanged in 13 (6.9%) patients, and worsened in 9 (4.8%) patients (Table 6).

## 4. Discussion

Although uveitis is less common in children than in adults [1], the rate of visual impairment in children secondary to the complications of uveitis is higher than in adults [6]. This makes the accurate and early diagnosis of uveitis in children critical. There are many causes of uveitis, and there are regional and ethnic differences in causes of uveitis. Familiarity with the distribution of local causes of uveitis should help doctors to accurately diagnose this disease.

Few studies have focused on pediatric uveitis in China. A previous study describing the clinical patterns of uveitis in a tertiary referral center in North China, which included the pediatric uveitis group and found that 21.0% of cases were in children under the age of 16 years [28]. The present study is the first one to specifically focus on the clinical profile of pediatric uveitis in East China, revealing a predominance of chronic (62.9%) and noninfectious cases (81.3%), with anterior involvement (54.8%).

Of the patients involved, more were > 8 years old (67.1%) than were ≤ 8 years old (32.9%). The different stages of immune system development, differences in environmental exposure, and the natural history of the diseases may explain this difference [29]. Moreover, because it is usually difficult for young children to express their symptoms verbally, the disease may only be identified sometime after its onset, thus delaying a diagnosis [30].

The disease distribution characteristics of pediatric uveitis also differ at different ages. Pediatric uveitis was slightly more common in girls (51.6%) in the present study, which is similar to previously reported studies (53.1% in the United States, published in 2009; 70.3% in Japan, published in 2016; 53% in Finland, published in 2020) [3, 5, 8]. This was more evident in patients younger than 8 years, in which the proportion of girls was 62.4% in the present study. However, in the 9–16-year age group, boys were diagnosed with uveitis more frequently than girls (53.7% vs. 46.3%, respectively). This is also consistent with a previous study investigated in Lebanon and published in 2019, with a boy versus girl ratio of 35.3% versus 64.7% in the 0–8-year age group, and 50.0% versus 50.0% in the 9–16-year age group [30].

In patients aged 0–8 years, the most common types of uveitis were JIA-associated anterior uveitis (25 patients, 26.9%), idiopathic chronic anterior uveitis (21 patients, 22.6%), and ocular toxocariasis (17 patients, 18.3%), whereas in patients aged 9–16 years, the most common types were idiopathic acute anterior uveitis (42 patients, 22.1%), idiopathic chronic anterior uveitis (28 patients, 14.7%), and ocular toxocariasis (25 patients, 13.2%). These differences in etiology in different age groups may explain the differences in the sex-based distributions. For example, younger girls are more likely to have JIA-associated anterior uveitis [31], whereas boys at an older age are more likely to suffer from some infectious uveitis, such as ocular toxocariasis [32].

Uveitis can be divided into noninfectious and infectious depending on the etiologies. In the present study, the most common diagnosis for noninfectious uveitis was chronic anterior uveitis (95 patients, 33.6% in total and 41.3% of those with noninfectious uveitis). The asymptomatic nature of chronic anterior uveitis and the difficulties entailed in comprehensively examining the eyes of children can delay diagnosis and treatment, increasing the risk of developing ocular complications in children with chronic anterior uveitis. Chronic anterior uveitis occurred in a heterogeneous population in the present study, with the idiopathic disease being the most common (49 patients, 17.3% in total and 21.3% of those with noninfectious uveitis) and JIA-associated anterior uveitis another important subgroup (46 patients, 16.3% in total and 20.0% of those with noninfectious uveitis). Some studies have indicated that idiopathic disease involves more severe inflammation, but whether this disease represents a distinct clinical entity requires further investigation [33]. Identifying the systemic features of the disease may sometimes be a challenge for ophthalmologists, who must keep in mind that the signs or symptoms of an underlying systemic disorder may not be apparent at the onset of uveitis. For example, anterior uveitis can occur before the onset of arthritis in patients with JIA [31].

The proportion of JIA-associated anterior uveitis among patients with chronic anterior uveitis varies from 38% to 80% [33–35]. In the present study, 48.4% of patients with chronic anterior uveitis had a diagnosis of JIA, consistent with previous studies. The frequency of JIA-associated anterior uveitis varies geographically and among ethnicities. It is the most common noninfectious uveitis in Europe and the United States, accounting for 20%–60% of pediatric uveitis [3, 5, 7], but is less frequent in Asian countries, such as India (11.6%) and Singapore (1.8%) [12, 13], and is even rarer in Japan, where no case has been reported in one study [8]. Few studies have focused on JIA-associated anterior uveitis in China. One study reported that the clinical patterns of uveitis in a tertiary referral center in North China indicated that 8.7% of pediatric uveitis was JIA-associated anterior uveitis [28], consistent with our observation. Differences in the awareness of JIA-associated anterior uveitis and access to health care may affect the reported incidence in certain regions, explaining the geographic variation [36]. Different genetic backgrounds may also affect the different geographic incidence of this disease, especially differences in the frequency of HLA haplotypes [37]. One study showed that the percentage of JIA patients who developed JIA-associated anterior uveitis was similar to that of European and those with non-European ancestry, which indicated that the different prevalence of JIA might be another explanation for the different prevalence of JIA-associated anterior uveitis in different countries and ethnic groups [36].

Infectious etiologies account for up to 13% of all childhood uveitis [38]. There are obvious differences in the pathogens that occur in different regions, which may be closely related to the environmental differences in different regions, the degree of urban development, and people's living habits. In developed countries, the noninfectious etiology predominates, whereas an infectious etiology is more common in developing countries.

Ocular toxoplasmosis is the most common form of infectious uveitis in children in most regions, including the United States (43.9% of those with an infectious etiology and 4.7% of all uveitis) [5], the Netherlands (46.5% of those with an infectious etiology and 34.5% of all uveitis) [39], Singapore (22.2% of those with an infectious etiology and 7.4% of all uveitis) [13], and Colombia (76.8% of those with an infectious etiology and 44.9% of all uveitis) [11]. Varicella zoster virus is the most common pathogen causing infectious pediatric uveitis in Finland (69.2% of those with an infectious etiology and 6.0% of all uveitis) [3], whereas in India, the most common diagnosis is ocular tuberculosis (31.8% of those with an infectious etiology and 7.4% of all uveitis) [12].

Ocular toxocariasis was the most common infectious etiology in the present study, occurring in 79.2% of those patients with an infectious etiology and in 14.8% of all patients with uveitis. The increasing rates of pet ownership, irregular veterinary care for the pets, and substandard living conditions all lead to exposure to soil and food contaminated with Toxocara ova shed in the feces of dogs and cats, which is believed to be the risk factors for infection with Toxocara in the rural area in East China. Children are at greater risk of ocular toxocariasis due to their patterns of play with pets [17]. A better understanding of ocular toxocariasis by ophthalmologists in these areas has increased the rate of diagnosis of this disease, which is another possible explanation for the higher reported incidence. Ocular toxocariasis is also common in several other developing countries, including India (18.2% of those with an infectious etiology and 4.2% of all uveitis) and Colombia (17.7% of those with an infectious etiology and 10.3% of all uveitis) [11, 12], which might share some similarities in the aspects of substandard living conditions and irregular veterinary care of the pets.

For children with uveitis, the focus of treatment is the successful control of inflammation and prevention of complications. At present, available drug treatments are increasing. In addition to traditional immunosuppressants, biological agents are used ever more widely, and all of these allow ophthalmologists to more easily control the inflammatory response of uveitis in children. When we analyzed the visual prognoses of the enrolled patients, we found that the visual acuity of patients who received personalized anti-inflammatory treatment improved to varying extents. However, among patients on the same anti-inflammatory regimen, those with complications requiring surgery showed less vision improvement than those who did not require surgery. Complications of pediatric uveitis may occur in the early phase of the disease and have lifelong sequelae for the visual function of patients [40]. This suggests that the early recognition and timely treatment of uveitis in children and the prevention of complications are important for their visual prognoses.

It must be mentioned that most patients who required surgery had longer follow-ups in the present study compared with the unoperated eyes, and the difference was statistically significant in the overall patients (*p*=0.001), the patients receiving local medical treatment (*p* < 0.001), and the patients receiving systemic immunosuppressive drugs (*p*=0.002). The difference in follow-up periods might cause bias when comparing visual outcomes between groups, which reminded interpreting the visual outcomes with caution. The difference in the follow-up period might be related to the severity of the patient's condition to some degree. Patients with milder conditions might be able to achieve better inflammation control after a shorter period of treatment and then were lost to follow-up after a relatively short follow-up period. The bias comes from the retrospective nature of the present study, and well-designed studies in the future are needed to draw more accurate conclusions.

The present study contributed to our knowledge of the clinical spectrum of pediatric uveitis in China, which should help ophthalmologists diagnose and manage children with uveitis. However, this study also had some limitations, including its retrospective design, the lack of standardized patient visits, and missing of some follow-up data. The review was conducted at a tertiary center, which might have biased the clinical patterns of the disease. Also, the analysis of visual outcomes was based on the eyes with the worst visual acuity, and this should be noted when comparing our data with other data published in the literature.

## 5. Conclusions

In conclusion, pediatric uveitis in this cohort was predominantly chronic and noninfectious, with anterior involvement. Systemic therapy was required by most patients, and the visual acuity of most eyes improved with treatment.

## Figures and Tables

**Table 1:** Demographic and clinical characteristics of pediatric patients with uveitis.

Location	Total (*n* = 283)		Anterior (*n* = 155)		Intermediate (*n* = 13)		Posterior (*n* = 43)		Panuveitis (*n* = 72)	
	*n*	(%)	*n*	(%)	*n*	(%)	*n*	(%)	*n*	(%)
Sex										
Male	137	48.4	69	44.5	5	38.5	19	44.2	44	61.1
Female	146	51.6	86	55.5	8	61.5	24	55.8	28	38.9
Age (years)										
≤ 8	93	32.9	52	33.5	6	46.2	11	25.6	24	33.3
> 8	190	67.1	103	66.5	7	53.8	32	74.4	48	66.7
Course										
Acute	83	29.3	53	34.2	0	0.0	20	46.5	10	13.9
Chronic	178	62.9	94	60.6	13	100.0	21	48.8	50	69.4
Recurrent	22	7.8	8	5.2	0	0.0	2	4.7	12	16.7
Laterality										
Unilateral	143	50.5	74	47.7	6	46.2	11	25.6	52	72.2
Bilateral	140	49.5	81	52.3	7	53.8	32	74.4	20	27.8
Etiology										
Infectious	53	18.7	0	0.0	0	0.0	7	16.3	46	63.9
Noninfectious	230	81.3	155	100	13	100.0	36	83.7	26	36.1
Classification										
Idiopathic	142	50.2	98	63.3	13	100.0	26	60.5	5	6.9
Ocular	61	21.6	3	1.9	0	0.0	6	13.9	52	72.2
Systemic	80	28.3	54	34.8	0	0.0	11	25.6	15	20.9

**Table 2:** Etiology of pediatric patients with uveitis (Part 1).

Infectious (*n* = 53)		Noninfectious (*n* = 230)			
Etiology	*n*	Etiology		*n*	
Toxocariasis	42	Chronic anterior uveitis	Idiopathic	49	95
Viral	4		JIA	46	

Toxoplasmosis	3	Idiopathic acute anterior uveitis			47

Tuberculosis	2	Idiopathic retinal vasculitis			19

Streptococcal	1	Idiopathic intermediate uveitis			13

Bartonellosis	1	Behçet's disease			12

		Others			44

Abbreviation: JIA, juvenile idiopathic arthritis.

**Table 3:** Etiology of pediatric patients with uveitis (Part 2).

Classification	Location	Etiology		Total	Sex		Age (years)	
					Male	Female	≤ 8	> 8
					*n*	*n*	*n*	*n*
Idiopathic (*n* = 142, 50.2%)	Anterior (*n* = 98)	Chronic		49	20	29	21	28
		Acute		47	24	23	5	42
		Recurrence		2	0	2	0	2
	Intermediate (*n* = 13)			13	5	8	6	7
	Posterior (*n* = 26)	Retinal vasculitis		19	7	12	4	15
		Choroiditis		6	3	3	2	4
		Neuroretinitis		1	1	0	1	0
	Panuveitis (*n* = 5)			5	2	3	2	3

Ocular (*n* = 61, 21.5%)	Anterior (*n* = 3)	Posner–Schlossman syndrome		3	3	0	0	3
	Posterior (*n* = 6)	MEWDS		2	0	2	0	2
		AZOOR		1	0	1	0	1
		Ocular toxoplasmosis		3	3	0	2	1
	Panuveitis (*n* = 52)	Ocular toxocariasis		42	28	14	17	25
		Vogt–Koyanagi–Harada disease		6	3	3	0	6
		Acute retinal necrosis		4	0	4	0	4

Systemic (*n* = 80, 28.3%)	Anterior (*n* = 54)	JIA		46	16	30	25	21
		B27+		6	5	1	0	6
		Sjögren's syndrome		1	0	1	1	0
		GVHD		1	1	0	0	1
	Posterior (*n* = 11)	Retinal vasculitis	SLE	4	1	3	0	4
			B27+	1	1	0	0	1
			Thrombotic microangiopathy	1	1	0	1	0
			Aplastic anemia	1	0	1	1	0
			Streptococcus	1	0	1	1	0
			Tuberculosis	1	1	0	0	1
		Neuroretinitis	Bartonellosis	1	1	0	0	1
		Choroiditis	Tuberculosis	1	1	0	0	1
	Panuveitis (*n* = 15)	Behçet's disease		12	8	4	2	10
		Blau syndrome		3	2	1	2	1

Abbreviations: AZOOR, acute zonal occult outer retinopathy; GVHD, graft-versus-host disease; JIA, juvenile idiopathic arthritis; MEWDS, multiple evanescent white dot syndrome; SLE, systemic lupus erythematosus.

**Table 4:** Medical treatment modalities and visual outcomes.

Treatment modalities			Location					Visual acuity			
Medical treatment		Surgical treatment	Total	Anterior	Intermediate	Posterior	Panuveitis	At presentation	At last follow-up	Follow-up (Months)	*p* valuea
Local treatment	Eyedrops/periocular (*n* = 82)	No	76	65	4	7	0	0.236 ± 0.324	0.098 ± 0.350	3.8 ± 2.1	< 0.001
		Yes	6	4	0	2	0	1.181 ± 0.521	1.172 ± 1.097	19.2 ± 17.7	0.893

Systemic treatment	Prednisone only (*n* = 62)	No	53	5	4	7	37	0.786 ± 0.577	0.279 ± 0.383	16.4 ± 12.1	< 0.001
		Yes	9	1	0	0	8	1.436 ± 0.452	0.938 ± 0.846	25.3 ± 18.2	0.109
	Immunosuppressive drugs^b^ (*n* = 83)	No	73	52	3	9	9	0.383 ± 0.402	0.148 ± 0.306	13.8 ± 10.3	< 0.001
		Yes	10	6	1	3	0	1.840 ± 0.885	0.764 ± 0.584	22.4 ± 6.7	0.028
		MTX (78); CsA (2); HCQ (1); MTX ⟶ CsA (1); sulfasalazine (1)									
	Biologic agentc (*n* = 44)	No	34	14	1	10	9	0.539 ± 0.449	0.343 ± 0.695	18.0 ± 13.8	0.002
		Yes	10	8	0	1	1	1.669 ± 1.142	0.942 ± 1.194	10.7 ± 10.6	0.069
		ADA (6); tofacitinib (2); MTX + ADA (25); MTX + infliximab (4); MTX ⟶ CsA + infliximab (1); MTX + mesalazine (1); MTX + infliximab + ADA (1); MMF + ADA (3); MMF + belimumab (1);									
	Anti-infective agentd (*n* = 12)	No	9	0	0	3	6	1.149 ± 0.338	0.308 ± 0.243	16.8 ± 6.6	0.043
		Yes	3	0	0	1	2	1.682 ± 1.246	0.647 ± 0.300	18.0 ± 15.9	0.109
		Antivirals (4); antituberculosis (2); anthelminthic (4); antibiotics (2);									

Abbreviations: ADA, adalimumab; CsA, cyclosporin A; HCQ, hydroxychloroquine; MMF, mycophenolate mofetil; MTX, methotrexate.

^a^Wilcoxon test.

^b^With or without prednisone administration.

^c^With or without prednisone/immunosuppressive drug administration.

^d^With or without prednisone administration.

**Table 5:** Surgical treatment modalities and visual outcomes.

Surgical treatment		Location					Visual acuity			
		Total	Anterior	Intermediate	Posterior	Panuveitis	At presentation	At the last follow-up	Follow-up (months)	*p* valuea
Cataract (*n* = 18)		18	12	1	2	3	1.896 ± 0.820	0.731 ± 0.876	19.9 ± 11.1	0.001
PPV (*n* = 9)	TRD (3);	9	0	0	2	7	1.696 ± 0.852	1.071 ± 0.758	23.3 ± 20.2	0.066
	TRD + ERM (1);									
	TRD + VH (1);									
	RRD (1);									
	ERM (2);									
	VH (1)									

Intravitreal injection (*n* = 5)	Anti-VEGF (3);	5	1	0	3	1	1.140 ± 0.194	1.624 ± 0.931	18.0 ± 12.7	0.223
	Ozurdex (2)									

Other (*n* = 11)	LPI/Iridectomy (*n* = 6); band keratopathy scraping (*n* = 5)									

Abbreviations: ERM, epiretinal membrane; LPI, laser peripheral iridotomy; PPV, pars plana vitrectomy; RRD, rhegmatogenous retinal detachment; TRD, tractional retinal detachment; VEGF, vascular endothelial growth factor; VH, vitreous hemorrhage.

^a^Wilcoxon test.

**Table 6:** Visual outcomes of patients with pediatric uveitis.

Visual acuity at presentation	Number of patients	Visual acuity at the last follow-up	Number of patients	Change in visual acuity	Number of patients
≤ 20/200	70/241	≤ 20/200	19/188	Improved	166/188
(20/200–20/50]	63/241	(20/200–20/50]	30/188	Stayed the same	13/188
> 20/50	108/241	> 20/50	139/188	Decreased	9/188

## Data Availability

The datasets generated and/or analyzed during the current study are available from the corresponding author on reasonable request.
